# Prospective Quantitative Neuroimaging Analysis of Putative Temporal Lobe Epilepsy

**DOI:** 10.3389/fneur.2021.747580

**Published:** 2021-11-05

**Authors:** Kost Elisevich, Esmaeil Davoodi-Bojd, John G. Heredia, Hamid Soltanian-Zadeh

**Affiliations:** ^1^Department of Clinical Neurosciences, Spectrum Health, Grand Rapids, MI, United States; ^2^Department of Surgery, College of Human Medicine, Michigan State University, Grand Rapids, MI, United States; ^3^Radiology and Research Administration, Henry Ford Health System, Detroit, MI, United States; ^4^Imaging Physics, Department of Radiology, Spectrum Health, Grand Rapids, MI, United States; ^5^Control and Intelligent Processing Center of Excellence (CIPCE), School of Electrical and Computer Engineering, University of Tehran, Tehran, Iran

**Keywords:** neuroimaging, temporal lobe epilepsy, MRI, lateralization, multimodal analysis

## Abstract

**Purpose:** A prospective study of individual and combined quantitative imaging applications for lateralizing epileptogenicity was performed in a cohort of consecutive patients with a putative diagnosis of mesial temporal lobe epilepsy (mTLE).

**Methods:** Quantitative metrics were applied to MRI and nuclear medicine imaging studies as part of a comprehensive presurgical investigation. The neuroimaging analytics were conducted remotely to remove bias. All quantitative lateralizing tools were trained using a separate dataset. Outcomes were determined after 2 years. Of those treated, some underwent resection, and others were implanted with a responsive neurostimulation (RNS) device.

**Results:** Forty-eight consecutive cases underwent evaluation using nine attributes of individual or combinations of neuroimaging modalities: 1) hippocampal volume, 2) FLAIR signal, 3) PET profile, 4) multistructural analysis (MSA), 5) multimodal model analysis (MMM), 6) DTI uncertainty analysis, 7) DTI connectivity, and 9) fMRI connectivity. Of the 24 patients undergoing resection, MSA, MMM, and PET proved most effective in predicting an Engel class 1 outcome (>80% accuracy). Both hippocampal volume and FLAIR signal analysis showed 76% and 69% concordance with an Engel class 1 outcome, respectively.

**Conclusion:** Quantitative multimodal neuroimaging in the context of a putative mTLE aids in declaring laterality. The degree to which there is disagreement among the various quantitative neuroimaging metrics will judge whether epileptogenicity can be confined sufficiently to a particular temporal lobe to warrant further study and choice of therapy. Prediction models will improve with continued exploration of combined optimal neuroimaging metrics.

## Introduction

Optimization of the process of lateralizing mesial temporal lobe epilepsy (mTLE) through a quantitative analysis of an assortment of imaging applications has been pursued for over 25 years. Clinical decision-support mechanisms enhanced by artificial intelligence have the ability to refine decision making by rendering greater objectivity to investigation. By doing so, clinical pathways may be shortened and better outcomes secured by better informed surgical applications. The selection of patients for surgical candidacy in cases of a putative mTLE has involved a number of neuroimaging metrics applied, in particular, to the hippocampus. Several publications have addressed quantitative MRI applications such as hippocampal volumetry (1–6) and signal intensity measures pertaining to T2-weighted and fluid-attenuated inversion recovery (FLAIR) analyses (7–15). Additional MRI applications have involved texture analysis (10, 16, 17), diffusion weighting and diffusion tensor image (DTI) analysis (18–25) and resting-state functional (rsf) MRI study (26–34). Both SPECT (35–43) and PET (44–46) have provided further confirmation of focal epileptogenicity in select cases and have proven beneficial in surgical decision-making. It is clear that TLE brings about hemispheric changes that manifest as derivative effects of network-generated activity. We take advantage of these wide-ranging changes through the application of a variety of neuroimaging methods to decide upon the laterality of expression. The degree of indeterminacy and/or discordance of these measures may underlie the likelihood of establishing a definitive laterality and achieving success with a targeted resection within the temporal lobe.

The abundance of neuroimaging studies prompts the question of what their cumulative impact may be upon the lateralization of a temporal epileptogenicity or whether the condition may be declared as residing wholly or partially in the temporal lobe at all. Imaging modalities naturally differ in their reliability and may, to some degree, show discrepancies in predicting laterality. Prior individual analyses of such studies have been undertaken with retrospective data to determine the ability to affirm laterality in cases of known mTLE (22, 47–51). The use of hippocampal volumetric and FLAIR signal intensity combined with SPECT-derived signal intensity (i.e., SISCOM), in particular, provided accurate lateralization in patients who had previously required intracranial electrographic confirmation prior to resection (51).

Each imaging technique provides only a limited perspective upon the condition and each has inherent weaknesses in its respective application (52). Ostensibly, a multimodal imaging approach would enhance lateralization capability by accruing data, which, if concordant, would provide the required confirmation to proceed with either intracranial electrographic study targeting the appropriate area or to proceed directly with resective surgery in those cases where electrophysiological attributes were in agreement. As such, a multimodal integrative approach could reduce the magnitude of the required surgical exposure and perhaps, in some cases, even forego the need for invasive monitoring (53).

This study seeks to establish what opportunities exist in the use of a multimodal quantitative neuroimaging approach in defining the laterality of a putative mTLE or identifying whether an epileptogenic network is likely to be fully or partially resident within the mesial temporal structure or at all. In those cases where resection was performed, outcome measures (i.e., Engel classification) were used to establish the accuracy of the decision-making process as it was carried out in current standard fashion as well as that by various neuroimaging metrics. Likewise, an assessment was made of the presence of indeterminacy by individual or combinations of neuroimaging attributes and/or the degree of discordance among these attributes to judge whether, in fact, a unilateral epileptogenicity existed in the temporal lobe.

## Methods

### Data

Neuroimaging data for the prospective study population of epilepsy patients was acquired from a single healthcare system. Datasets were obtained using clinical standard-of-care protocols and according to needs dictated for each patient. Hence, not all studies were performed for all patients although a number of studies were performed in the majority of patients and are identified accordingly. All MRI data were acquired using 3D T1-weighted and FLAIR imaging protocols regardless of imaging parameters. Certain cases also had included high-resolution DTI, rsfMRI, SPECT, and/or PET data. A dataset, from Henry Ford Hospital (HFH, Detroit MI), consisted of 133 mTLE patients who underwent resection and remained seizure-free after 24 months and 27 non-epileptic subjects as controls. The study was conducted with the approval of the Institutional Review Board (IRB) of the Henry Ford Health System (HFHS). This dataset was used only to train our lateralization prediction models. Non-epileptic subjects were used as the control group only in the *Hippocampal volumetry* section, *Hippocampal fluid-attenuated inversion recovery intensity* section, and *Diffusion tensor image uncertainty analysis* section below.

The second dataset, from Spectrum Health (SH, Grand Rapids MI), consisted of 48 prospective epilepsy patients. The study was approved by the IRB at SH as part of a comprehensive study format allowing for inclusion of participants in the surgical epilepsy program. Patients undergoing study for a putative mTLE were accrued over a period of 3 years to determine the impact of quantitative neuroimaging on establishing laterality. A cohort of 48 patients (16M:32F) with a mean age of 40 years (range 16–75) were acquired after ictal semiology and preliminary scalp electroencephalographic (EEG) study suggested a possible mTLE origin.

All patients presenting with features suggesting the presence of TLE were admitted consecutively into the study after consent was obtained to do so. Clinical assessment first addressed putative temporal ictal origins with semiological traits that included but were not limited to experiential and/or other sensory phenomena with a loss of consciousness, oroalimentary automatisms, and versive cephalo-ocular deviation with tonic posturing. A standard investigation followed with inpatient video-EEG monitoring over a minimum 5-day period as a Phase I study with MRI, PET, sodium amobarbital study, neuropsychological profile and, in several cases, magnetoencephalography (MEG). SPECT was used in just two cases at a time when the application was under development; hence, it was not included in the assessment. Sodium amobarbital study was completed in most subjects. In those cases where discordant features arose, particularly dealing with laterality and epileptogenic origin, a Phase II study was performed. The latter was undertaken with surface electrode arrays and/or multicontact depth electrodes targeting areas of concern in one or both cerebral hemispheres. In some of these situations, a Phase III study was warranted and involved a further introduction of surface and/or depth electrode arrays to better define an origin or extent of an epileptogenicity.

Several neuroradiologists provided qualitative impressions of the appearance of the mesial temporal structure in each patient. The reports were reviewed and all MRIs reexamined to render a final impression of MTS according to the following criteria: (1) reduction of hippocampal volume as determined by a right–left asymmetry on T1-weighted coronal images, (2) increased FLAIR signal intensity, and (3) loss of intrinsic hippocampal laminar structure. These features were required on at least two sequential coronal images to declare the impression of MTS.

### MR Imaging

Scans were acquired with a 1.5T and a 3T MRI system (GE Medical Systems, Milwaukee, WI) although over 93% of studies were performed by the latter. The pulse sequence parameters were not the same for the 1.5T and 3T MRIs because of differing field strengths. Both sets of imaging parameters were, however, set to optimize contrast for each scan, so that any contrast mismatch would be minimized between the two field strengths. We have previously shown no statistically significant difference between average amygdalar volumes of control subjects scanned with either 1.5T or 3T MRI units (*p* = 0.11) (54). Similarly, no statistically significant difference was identified between amygdalar FLAIR features of control subjects scanned with either 1.5T or 3T MRI units (*p* = 0.19 and *p* = 0.38 for amygdalar FLAIR mean and standard deviation ratios, respectively). All scanners had passed American College of Radiology (ACR) testing requirements to mitigate shifts in signal intensity inhomogeneity. A 3D T1-weighted MR study protocol consisted of a spoiled gradient echo (SPGR) sequence with TR/TI/TE = 7.6/1.7/500 ms, flip angle = 20°, field of view (FOV) = 200 × 200 mm^2^, matrix size 256 × 256, pixel size = 0.781 × 0.781 mm^2^, slice thickness = 2.0 mm (voxel size = 0.781 × 0.781 × 2.0 mm^3^), number of slices = 124, bandwidth 25 kHz, and scanning time of 5 min and 45 s. Coronal FLAIR MR datasets were acquired with TR/TI/TE = 10,002/2,200/119 ms, flip angle = 90°, FOV = 200 × 200 mm^2^, matrix size = 256 × 256, pixel size = 0.781 × 0.781 mm^2^, slice thickness = 3.0 mm (voxel size = 0.781 × 0.781 × 3.0 mm^3^), minimum number of slices = 47, bandwidth 20.8 kHz, and scanning time of 12 min. Image slices were acquired contiguously without gaps in all studies. When required, translational corrections were made to correct for head position from slice-to-slice, to avoid abandoning a study secondary to head motion. Most adjustments were made in the dorsoventral direction. Diffusion tensor images (*b*-value of 1,000 s/mm^2^) along with a set of null images (b-value of 0 s/mm^2^) were acquired using echo planar imaging (EPI) (55, 56) with TR/TI/TE = 7,500/0/76 ms, flip angle = 90°, voxel size = 1.96 × 1.96 × 2.6 mm^3^, imaging matrix 128 × 128, FOV of 240 × 240 mm^2^ and 25 diffusion gradient directions. Resting state fMRIs were acquired using a gradient echo EPI sequence with an FOV of 240 × 240 mm^2^ on a 64 × 64 matrix (voxel size = 3.75 × 3.75 × 4 mm^3^), and TR/TE = 2,000 ms/30 ms. Functional MRIs were taken at 2-s intervals for capture of activity while subjects lay quietly with eyes open within the scanner. In total, 150 volumes were recorded, each over 5 min.

### Quantitative Neuroimaging Analysis

Quantitative studies of MRI, specifically T1-weighted hippocampal volumes, FLAIR mean signal intensity with standard deviation, DTI connectivity measures of hippocampal-orbitofrontal and thalamo-temporopolar projections, and rsfMRI were performed. Additional analyses involved signal intensity measures with SPECT and PET. This battery of quantitative neuroimaging measures was performed remotely (Imaging Research Laboratory, HFH), while qualitative interpretations of these studies were performed at the primary institution (SH) as part of the standard investigation of a prospective surgical candidate. Data for single classifiers (i.e., hippocampal volumetry, FLAIR, DTI) were arranged on scatter plots and a boundary domain containing the uncertainty range was depicted using the classifier model. The classifier developed in each single modality model was optimized independent of other modalities. Therefore, the features and the optimized classifier are different among the single modality models.

All classifiers are developed from historical data from within our working group and relate to independent retrospective studies of individual quantitative metrics published over the past decade. These studies dealt with cohorts of patients who had attained Engel class 1 outcomes following standard clinical assessments of their TLE with non-epileptic control patients defining a boundary domain centrally within the scatter plots generated.

Within the hippocampal volume and FLAIR plots, boundary lines for each scatter plot were depicted parallel to the line fitted to the control measures with a 2sd margin allotted. When results were plotted beyond this margin, an “R” or “L” designation was applied to indicate a high confidence in laterality. In cases where there was a tendency toward right or left but with results plotted between the 1sd and 2sd lines, a designation of “UR” or “UL”, respectively, was applied to indicate a trend in laterality. When the result appeared within the 1sd boundary domain, the designation was “U” to indicate indeterminacy.

#### Hippocampal Volumetry

The regions of interest (ROIs) encompassing the hippocampi were outlined manually for segmentation using sequential coronal T1-weighted MR images with reference to an MRI atlas identifying the hippocampus (57). The accuracy of current automatic hippocampal segmentation techniques was judged insufficient to reliably avoid contamination of the FLAIR signal intensity measure with unwanted signal from outside the intended ROI, despite acceptable performance for hippocampal volume (2, 58, 59). Manual segmentation was used to minimize such error.

For volumetric analysis, the entire hippocampus was examined. The volumes in each case were calculated by summing the voxels occupying hippocampal ROIs and multiplying by voxel volume. Hippocampal volumes were normalized to the intracranial volume to reduce between-subject variability of the brain. Therefore, two features were calculated from hippocampal volumes:


(1) $$\begin{array}{l} f_{1}=\frac{Vol_{Left}^{Hipp}}{Vol^{Brain}}\times 100 \end{array}$$

(2) $$\begin{array}{l} f_{2}=\frac{Vol_{Right}^{Hipp}}{Vol^{Brain}}\times 100 \end{array}$$

#### Hippocampal Fluid-Attenuated Inversion Recovery Intensity

The method of Kim et al. (6) was used to establish anterior and posterior boundaries for hippocampal FLAIR analysis to reduce the impact of partial volume effect (6). The anterior coronal boundary plane coincided with the gyrus intralimbicus, whereas the posterior plane was the most caudal section through the quadrigeminal plate. Non-brain tissues were removed from T1-weighted and FLAIR images by manual segmentation. This improved the accuracy of subsequent coregistration. The skull-stripped T1-weighted and FLAIR image sets were coregistered using a rigid registration technique (FLIRT) (60) based on mutual information. The manually segmented ROIs were mapped onto the FLAIR MRI using the registration transform and then used for feature extraction. Inaccurate hippocampal segmentation and coregistration can result in misallocation of ROIs on corresponding FLAIR images resulting in the inclusion of unwanted sites containing disparate signal intensities (i.e., cerebrospinal fluid) and affecting estimates. To this end, the segmentation and coregistration outcomes were cross-checked using Eigentool. Specifically, new ROIs were placed on brain landmarks of coregistered T1-weighted images. These were then loaded into the FLAIR image dataset and rechecked for correct placement. No misalignment was found between coregistered T1-weighted and FLAIR images. Both a mean and standard deviation (SD) of FLAIR signal intensity were extracted from each hippocampal FLAIR intensity. The final value for each feature was expressed as a ratio of measured values of the two hippocampi for normalization purposes to avoid the problem of variance in FLAIR signal intensity from case-to-case and scan-to-scan, lessen the effect of imaging imperfections, and facilitate the classification and graphical analysis of left- and right-sided epileptogenicity.


(3) $$\begin{array}{l} f_{3}=\frac{meanFLAIR_{Left}^{Hipp}}{meanFLAIR_{Right}^{Hipp}} \end{array}$$

(4) $$\begin{array}{l} f_{4}=\frac{stdFLAIR_{Left}^{Hipp}}{stdFLAIR_{Right}^{Hipp}} \end{array}$$

The FLAIR scatter plot created shows SD of signal asymmetry vs. mean signal asymmetry.

#### Multistructural Analysis (MSA)

We have developed a decision tree-based classifier using multistructural analysis of preoperative T1-weighted images in a retrospective cohort of 68 TLE patients with an Engel class 1 surgical outcome (61, 62). The classifier uses the normalized volume changes [i.e., 100(vL – vR)/(vL + vR)] of the hippocampus, amygdala and thalamus and could lateralize mTLE with an accuracy of 98.5% using training data. For each patient, the brain structures were segmented automatically using FreeSurfer (S22). A boundary domain was determined with a logistic function model to identify the degree of certainty with which to distinguish laterality by a probability measure. If the probability equaled 0.5, the index case was labeled as “U” and, otherwise, if less than 0.7, labeling was UL or UR, designating lesser certainty of laterality then if it appeared outside the boundary domain.

#### Positron Emission Tomography Profile Analysis

Hypometabolic features were investigated only within the hippocampal region. For each patient, the 3D Standard Uptake Value (SUV) image, acquired from PET imaging, was skull-stripped (BET) and coregistered (rigid-FLIRT) to the skull-stripped T1-weighted MRI using the FSL tool. Then, for each subject, the hippocampal SUVs were extracted from the coregistered PET modality using the hippocampal ROIs. From anterior to posterior, SUV profiles for left and right hippocampi were extracted and their coronal sections compared with a test of significance (Welch test - unpaired and unequal variances) at a confidence level of 95%. Based on the result of the test, a measure for hypometabolism asymmetry (HA) was calculated for each coronal section. If SUVs of the left were significantly smaller than the right, then HA = (SUVl-SUVr)/SUVr; if SUVs of the right were significantly smaller than the left, then HA = (SUVl-SUVr)/SUVl; and, if there was no significant difference, HA was set to 0. Therefore, HA values ranged from −1 to 1, providing a laterality measure for each slice through the hippocampus with either left (HA < 0) or right (HA > 0) preference depending upon the extent of hypometabolism (see **Figures 2D**, **3D**; HA values are shown as a percent and color-coded with blue for HA < 0 and red HA >0). Such a profile can be used to designate the hypometabolic changes extending through the head, body, and tail segments of the hippocampus. The final laterality was summarized in a measure with −1 identifying a left-sided certainty and 1, a right-sided certainty for epileptogenicity [see Ref. (63) for details].

#### Multimodal Model Decision Scheme

As another approach toward establishing a greater refinement in distinguishing laterality of epileptogenicity, a combination of classifiers was used based upon a previous dataset of a specified number of classifiers. Each classifier with its laterality declared as L, R, UL, UR, or U was combined with others using a majority voting scheme (65). Since any patient may not have undergone all imaging modalities (i.e., T1-weighted MRI, FLAIR MRI, PET, or SPECT), we defined the rules for this model based upon various combinations of the following:

- Volumetry (V): Hippocampal volume lateralization outcome (R, UR, U, UL, L)
- MSA (M): Multistructural lateralization outcome (R, L)
- FLAIR (F): Hippocampal FLAIR lateralization outcome (R, UR, U, UL, L)
- SPECT (S): SPECT lateralization outcome (R, L)
- PET (P): PET profile analysis lateralization outcome (R, UR, U, UL, L)

Patients in this study had two to five classifier outcomes. The rules of the majority voting system combining these outcomes were as follows:


$$\begin{array}{l} Multimodal\;decision\;is\;X\;if \end{array}$$

(5) $$\{\begin{array}{l} 1.{\left(X\right)}^{3}{\left(.\right)}^{n=0,1,2}\;\\ 2.\;{\left(X\right)}^{2}{\left(.\right)}^{n=0,1}\;\\ 3.\;{\left(X\right)}^{2}{\left(.\right)}^{n=0,1}{\left(U.\right)}^{n=1,2}\;\\ 4.{\left(X\right)}^{1}{\left(UX\right)}^{n=1,2,3,4}{\left(U\right)}^{n=0,1}\; \end{array}and\;is\;U,\;if\;otherwise$$

where *X* denotes either a L or R epileptogenicity, (.) denotes any outcome that a single classifier can have (i.e., L, UL, U, UR, R) and the *power* indicates the number of outcomes for that decision. For example, a decision set of (L)(UR)(U)(L)(L) is summarized as (*X*)^3^(.)^2^. Here, rule 1 is applied. Rules 1 and 3 do not apply to decision sets with two outcomes and rule 2 does not apply to decision sets with five outcomes. The majority voting score was calculated using the following equation:


(6) $$\begin{array}{l} vs=\frac{\sum \left(X\right)+0.5\sum \left(UX\right)}{\sum \left(.\right)} \end{array}$$

in which ∑ denotes the summation. For example, the voting score of a decision set of (L)(UR)(U)(L)(L) amounts to 3/5 = 0.6.

#### Diffusion Tensor Image Uncertainty Analysis

DTI involves consideration of a high variability in diffusion indices. Hence, an analysis of hemispherical asymmetry of any bilaterally situated structure must show a significant result to exceed a certain hemispheric variation uncertainty (HVU) in order to be interpreted correctly. Using the DTI of 23 non-epileptic subjects and 20 TLE patients who had undergone surgical resection with Engel class 1 outcomes, we developed a prediction model for the laterality of seizure onset based on HVU levels of mean diffusivity (MD) in the hippocampus and fractional anisotropy (FA) in the posteroinferior cingulum and crus of the fornix (22). The variability in asymmetry was estimated using the same scanner and imaging parameters for both controls and patients. A higher hippocampal MD and lower posteroinferior cingulate and forniceal crus FA was identified ipsilateral to the side of seizure onset in 10/10 pathologically proven MTS cases.

The model involves the following tasks: (1) DTI preprocessing including isotropic voxel size resampling (1.96 × 1.96 × 1.96 mm^3^), diffusion tensor estimation and diffusion parameter calculation, (2) seed placement upon fiber bundles using color-coded DTI with an in-house developed user-interactive MATLAB-based tool, (3) fiber bundle segmentation and tractography, (4) FA and MD calculation for the segmented tracts, and (5) uncertainty analysis to predict seizure onset [refer to Ref. (22) for more details].

#### Diffusion Tensor Image Connectivity Analysis

For DTI connectivity analysis, we developed a support vector machine (SVM)-based classifier for lateralizing seizure onset (24). Brain regions were segmented on T1-weighted MRIs using FreeSurfer and fiber bundles extracted from DTI using MRtrix. Having defined whole fibers and the labeled segmented image, a connectivity matrix was built for each subject by calculating the connectivity strength between each pair of regions (i,j), i,jϵ 1,…,164. Two measures were used to calculate DTI connectivity strength: (1) Number-of-Fibers-based Connectivity, ***NFC***(i,j), derived by counting the number of fibers passing through both regions or connecting the two regions and normalizing to the total number of fibers passing through either of the two regions. (2) FA-based Connectivity, ***FAC***(i,j), calculated by averaging the mean-FA value of all fibers passing through or connecting the two regions. The mean FA value of each fiber is also derived by averaging the FA values of the voxels it passes through. These two measures reveal complimentary aspects of connectivity.

The white matter connectivity matrices of 73 epilepsy patients (18 extratemporal, 16 multifocal, 19 right temporal, 20 left temporal) were used to build a decision-making system employing two SVM classifiers. Classifier #1 discriminated TLE patients from others (*categorization*) and classifier #2 discriminated L-TLE from R-TLE subjects (*lateralization*). A prospective patient was identified if the evaluation of classifier #1 was TLE and the evaluation of classifier #2 was L or R. These two classifiers were trained using the approach described by Davoodi-Bojd et al. (24). We used an initial set of 1,600 fiber connections between regional pairs for feature selection.

The algorithm selected three features:


(7) $$\{\begin{array}{ccc} F1 & = & FAC\left(L\;parietal\;superior\;gyrus\right)\;\\ F2 & = & NFC(L\;ventral\;DC,\;L\;marginal\;cingulate\;sulcus)\\ F3 & = & NFC\left(L\;hippocampus,\;L\;ventral\;DC\right)\; \end{array}$$

for classifier #1 (categorization), and three features:


(8) $$\{\begin{array}{ccc} F4 & = & NFC\left(hippocampus\right)\;\\ F5 & = & FAC(amygdala,\;lateral\;superior\;temporal\;gyrus)\\ F6 & = & FAC\left(putamen,\;inferior\;temporal\;sulcus\right)\; \end{array}$$

for classifier #2 (lateralization), in which **Δ** refers to the connectivity difference between left and right hemispheres [i.e., F6 refers to *FAC* (*left putamen, left inferior temporal sulcus*)—*FAC* (*right putamen, right inferior temporal sulcus*)].

#### Resting-State Functional MRI Connectivity Analysis

This measure was applied in only four cases but has been added here to provide some comparison with the more standard measures identified above. Similar to DTI connectivity analysis (24), we have developed an SVM-based classifier for lateralizing epileptogenic onset. Brain regions from T1-weighted MRIs were segmented using FreeSurfer and labeled to 82 regions per hemisphere and then coregistered to the fMRI space. The rsfMRI data was processed using FSL, which includes eliminating the first five volumes due to magnetization equilibrium, brain extraction, motion correction, slicing timing, temporal high-pass filtering, and spatial smoothing (FWHM = 5 mm) and connectivity measures calculated as the Pearson's correlation between the temporal rsfMRI signals of each pair of the 164 regions. This resulted in a 164 × 164 connectivity matrix for each subject. The connectivity matrices of six left-TLE and five right-TLE patients were used to develop a linear SVM classifier for lateralizing TLE patients. The developed classifier used connectivity features of only two regions: (1) “right inferior frontal sulcus (IFS)” and (2) “right nucleus accumbens (NAc)”, with 100% accuracy achieved over the training datasets. The overall connectivity of these two regions was shown to be more reduced in right TLE than in left TLE patients. The developed rsfMRI classifier was used to identify the epileptogenic hemisphere of the prospective TLE patients.

### Data Analysis

Information regarding measures, particularly the methodologies related to each attribute, is described within the respective sections above. These relate to scatter plot analyses, boundary definitions, classifier schemes, and measures of significance within border domains. Further definition of these is given in the introduction to Table 1 below. The use of a multimodal model decision scheme considers the role of previous experience using some of the same attributes as in the current study and is subject to the shortfalls of retrospective review and the limitations regarding the particular attributes in use in the model, as with the inclusion of SPECT which was not implemented in the current study.

**Table 1:** Quantitative neuroimaging and clinical attributes of cases.

**Patient ID**	**Gender**	**Age** **(Surgery)**	**Duration**	**PhI**	**PhII/III**	**MTS (L/R/N)**	**Proc Side**	**12 Engel**	**24 Engel**	**(i) Vol Ratio**	**(ii) FLAIR (decision, mean, STD)**	**(iii) MSA**	**(iv) PET**	**(v) MMM**	**(vi) DTI Uncertainty**	**(vii) DTI Connectivity**	**(viii) fMRI Connectivity**
P01	M	35	11	F7>F3, T3	Lmt	N	L	3A	2C	UR 0.92	R (1.04, 0.97)	R	_	R 0.833	_	_	_
P02	F	NA	55	F8, F10	_	L	_	_	_	L 1.99	L (0.90, 0.82)	L	_	L 1.000	_	_	_
P03	F	NA	32	F3, F1>T3	_	R	R	2B	2B	R 0.73	R (1.08, 1.30)	UL	R	R 0.750	U	U	_
P04	F	32	25	F3, F7>T3	Lt	N	_	_	_	U 0.97	L (0.92, 0.92)	L	L	L 0.750	_	_	_
P05	F	NA	59	F7, T3	_	L	L	1A	1A	L 1.31	L (0.70, 0.54)	L	_	L 1.000	_	_	_
P07	M	NA	17	F7, T1, T3	_	L	L	2A	3A	L 1.39	U (1.00, 0.96)	L	_	L 0.667	_	_	_
P08	F	NA	10	T1, T5, T6	_	N	_	_	_	U 0.99	R (1.05, 1.08)	UR	_	R 0.500	L	U	_
P09	F	NA	61	T3, F7	_	L	_	_	_	L 1.69	L (0.91, 0.88)	L	_	L 1.000	U	L	_
P10	M	NA	24	T2, F8	_	R	R	1A	1A	R 0.84	R (1.06, 1.06)	R	R	R 1.000	U	R	_
P11	F	19	8	T1, F7, T3	Lmt	N	L	1A	1A	UL 1.08	UL (0.98, 1.02)	L	_	L 0.667	_	_	_
P12	M	16	6	F7, T1>T3	Lmt	L	L	1A	1A	UL 1.09	L (0.96, 0.87)	L	L	L 0.875	L	_	_
P14	F	NA	4	F7, T3	_	N	_	_	_	UL 1.09	L (1.17, 1.16)	L	_	L 0.833	U	U	_
P15	F	NA	59	T3>F7	_	L	L	1A	1A	L 2.09	L (0.91, 0.82)	L	L	L 1.000	L	_	_
P16	M	34	17	T4, F8	Rtp	N	R	1A	1A	R 0.87	R (1.07, 1.07)	R	L	R 0.750	_	_	_
P17	F	65	49	T4, F8	Rmt	N	R	1A	1A	U 0.97	R (1.06, 1.15)	U	_	R 0.333	U	U	_
P18	F	NA	3	T3>T1	_	L	L	1A	2A	R 0.9	L (0.95, 0.91)	UR	L	L 0.500	U	L	L
P19	M	43	17	T1>T3	Lmt	N	L-RNS	_	_	U 0.96	_	UR	UL	U	L	L	_
P20	F	32	5	T2>F8	Rmt	N	R	1A	1A	UL 1.1	UR (1.01, 0.95)	R	U	U	U	UL	_
P21	F	44	33	F7>T1, T2>T4	Lmt	N	B-RNS	_	_	U 0.95	UL (0.98, 0.84)	UR	L	U	U	L	_
P22	F	23	1	T2, T4	Rmt	N	R	3A	2B	UL 1.10	U (1.01, 1.01)	UR	U	U	L	U	_
P23	F	33	7	T3>T1	Lt>Rt	N	_	_	_	UL 1.07	L (0.85, 0.82)	UR	L	L 0.625	L	R	_
P24	M	53	20	T1, T3>F7	Ltc	N	L-RNS	1B	1B	U 1.00	L (0.91, 0.90)	UR	L	L 0.500	U	U	_
P25	F	NA	2	T1, T3>F7	_	N	_	_	_	L 1.14	R (1.03, 1.44)	F	UR	U	_	_	R
P26	F	NA	7	C4-P4, T4-T6	_	N	_	_	_	U 1.00	R (1.04, 0.76)	R	_	R 0.667	_	_	R
P27	F	65	64	F7	Lmt>Rtc	L	L	1A	1A	L 1.74	_	L	L	L 1.000	L	U	_
P28	F	41	37	C3-P3	Lf	N	L-RNS	_	_	UL 1.07	U (1.01, 0.97)	R	_	U	_	_	_
P29	F	38	33	T5>F7, T3	Lmt	N	L	2B	1B	R 0.66	R (1.13, 0.71)	R	L	R 0.750	L	L	_
P30	M	58	14	T2>F8, T4	Rmt	N	R	1A	1A	UR 0.90	R (1.13, 1.15)	R	R	R 0.875	U	R	_
P31	M	NA	64	F8, O2	Rmt	R	R	1B	1A	R 0.62	_	R	R	R 1.000	_	_	_
P32	F	44	24	F8>T2,T6>O2	Rmt, Rbto	N	R	1A	1A	UL 1.06	U (1.00, 0.82)	R	R	R 0.500	_	U	_
P33	M	73	1	C4, P4, T6	Rmt	R>L	R	3A	3A	R 0.46	UL (0.96, 1.07)	R	_	R 0.667	_	_	_
P34	M	44	40	F8, T8	Rstg, Rph	N	R-RNS	_	_	UL 1.11	L (0.96, 1.11)	R	_	U	L	F	F
P35	M	50	5	F7, T1, T2, T8	Lmt>Rmt	N	B-RNS	_	_	U 0.98	UL (0.98, 1.04)	UL	L	L 0.500	_	_	_
P36	F	50	53	F7>T1,T2,T4	Lmt>Rmt	N	B-RNS	_	_	U 0.92	U (1.00, 1.09)	R	L	U	U	L	_
P37	F	47	40	P8,D2,T7,F7	Lt	N	B-RNS	_	_	UL 1.10	_	L	L	L 0.833	_	_	_
P39	F	NA	6	N	_	N	_	_	_	L 1.23	U (0.99, 1.26)	L	_	L 0.667	_	L	_
P41	F	28	28	T7, C3, F8, T8	Lmto, Rp	N	B-RNS	_	_	R 0.78	L (0.93, 0.86)	R	R	R 0.750	_	F	F
P42	F	35	19	T7, F7	Lbto	N	L	3A	3A	UL 1.1	L (0.96, 1.05)	R	_	U	_	_	_
P43	F	32	5	T2, T8, F8	Rmt>Lmt	L	R-RNS	1A	1B	L 1.25	_	R	_	U	R	L	R
P44	F	43	43	Sp2	Rmt	N	R	1A	1A	R 0.75	_	R	_	R 1.000	_	_	_
P45	F	47	10	T1, F7, T7	Lbt, Lto	N	L-RNS	_	_	U 0.95	L (0.96, 1.10)	UL	L	L 0.625	_	_	_
P46	M	14	10	C3, C4	Lpc, Lph	N	L-RNS	_	_	U 0.96	_	R	L	U	U	UL	_
P47	M	62	10	F8, T8	_	R	R	2B	1C	R 1.39	_	R	R	R 1.000	R	R	_
P48	M	38	27	F3, F7, T1>T7	Lmt>Lmf	N	L	1A	1A	UL 1.15	U (0.99, 1.27)	R	_	U	UL	L	_
P49	F	65	44	T2, T9>F8, T8	Lmt>Lmf	N	B-RNS	_	_	U 0.96	_	R	_	R 0.500	_	_	_
P50	M	27	27	P8> P2	Rbt, Rpo	N	R-RNS	_	_	R 0.67	_	U	L	U	UR	L	_
P51	F	18	15	T3>F8, T2	Rmt	R	R	1A	1A	UR 0.81	UL (0.95, 1.86)	R	R	R 0.625	_	_	_
P52	F	19	12	T7, T9, P9	Ltc	N	L-RNS	_	_	U 0.97	U (1.00, 1.01)	R	L	U	_	_	_

*PhI: phase one EEG and video-EEG monitoring impression. PhII/III: intracranial EEG monitoring result. MTS: mesial temporal sclerosis side, left (L), right (R), or no MTS (N). Proc Side: side of operation, resection and/or RNS. 12 and 24 Engel: Engel class outcome after 12 and 24 months, resp. Vol Ratio: volumetry scatter plot and the L/R hippocampal volume ratio (f_1_). FLAIR (decision, mean, STD): FLAIR scatter plot with mean and STD FLAIR intensity ratios (f_3_ and f_4_, respectively). MMM: multimodal model voting result and score. Anatomical abbreviations: bt, basal temporal; bto, basal temporo-occipital; f, frontal; mt, mesial temporal; mto, mesial temporo-occipital; p, parietal; ph, parahippocampal; pc, posterior cingulate; stg, superior temporal gyrus; t, temporal; tc, temporal convexity; tp, temporoparietal*.

Comparison with the outcomes of our standard decision-making clinical protocol with those predicted by the various attributes and multimodal model implemented here is made in tabular form. These denote the accuracy of prediction of correct laterality as indicated by an Engel class 1 outcome in comparison with that in the case of an Engel class ≥2 outcome. A two sample independent *t*-test was applied to identify the extent of discrepancies among the individual quantitative metrics with standard clinical decision-making that yielded Engel class 1 outcomes *vs*. Engel class ≥2 outcomes.

## Results

### Patient Population

Case numbers P01 to P52 comprise the 48 study patients (16M, 32F). Cases P06, P13, P38, and P40 were excluded from the study because of incomplete workup and departure from the system. Of the remaining, 10 (21%) remain unoperated (P02, 04, 08, 09, 14, 23, 25, 26, 35, 39). Another 14 (29%) underwent RNS implantation (NeuroPace) after Phase II study definition of the site(s) of epileptogenicity (P19, 21, 24, 28, 34–37, 41, 43, 45, 46, 49, 52). Two of the latter group (P24, 43) underwent both a resection and RNS implantation at the conclusion of the Phase II study and were considered part of the RNS implant group as their epileptogenic network was not remediable by resection alone. Both attained Engel class 1 outcomes. The remaining 24 cases constitute the population of patients who had undergone resection, not all of whom had a standard temporopolar topectomy + amygdalohippocampal removal. One case was coupled with resection of an ipsilateral superior temporal convexity angioma (P17), while another (P48) had an additional remote epileptogenicity expressed in the ipsilateral mesiobasal frontal region at the site of an angioma. One case (P32) underwent an extended basal temporo-occipital resection and another (P42) manifested a discrete epileptogenicity in the basal temporo-occipital area where a cortical dysplasia was identified and a local resection carried out. A final case (P16) had abundant bilateral periventricular nodularity giving rise to other epilepsy types although the primary presenting epilepsy was effectively remediated by the standard resection mentioned above.

### Clinical Attributes

Table 1 presents the clinical attributes and quantitative neuroimaging results for each patient including Phase I, II, and occasional Phase III features, surgery type and laterality (R, L) with 12- and 24-month outcomes. For the single modality classifiers, uncertainty boundaries are defined as depicted in Table 2. “F” signifies a failure to segment a structure sufficiently well for measurement. For MMM, the decision is based on majority voting rules with a score assignment as shown in equations (5) and (6).

**Table 2:** Definition of decision boundaries and rules for single modality classifiers.

**Model**	**Features**	**Decision function**	**Decision boundaries**	**Decision rules**
Volumetry	Hippocampal volumes, f_1_ and f_2_	Linear (Scatter plot Eigenvector)	1 and 2 standard deviations (sd) off the decision line	(0,sd)→U (sd,2sd)→UL/UR (2sd,∞)→L/R
FLAIR	Hippocampal FLAIR intensities, f_3_ and f_4_	Linear (Scatter plot Eigenvector)	1 and 2 sd off the decision line	(0,sd)→U (sd,2sd)→UL/UR (2sd,∞)→L/R
MSA	Hippocampal, amygdalar, and thalamic volumes	Logistic function	0.2 sd off the decision line (i.e., probability equal to 0.5)	p=0.5→U 0.5 < p <0.7 → UL 0.3 < p <0.5 → UR p≥0.7 → L p ≤ 0.3 → R
DTI connectivity	Connectivity measures [equations (7) and (8)]	SVM	0.5 and 1 maximum margin of support vectors (M) off the decision line	(0,0.5M)→ U (0.5M,M)→ UL/UR (M,∞)→L/R

#### Phase I Study

**Figure 1:**
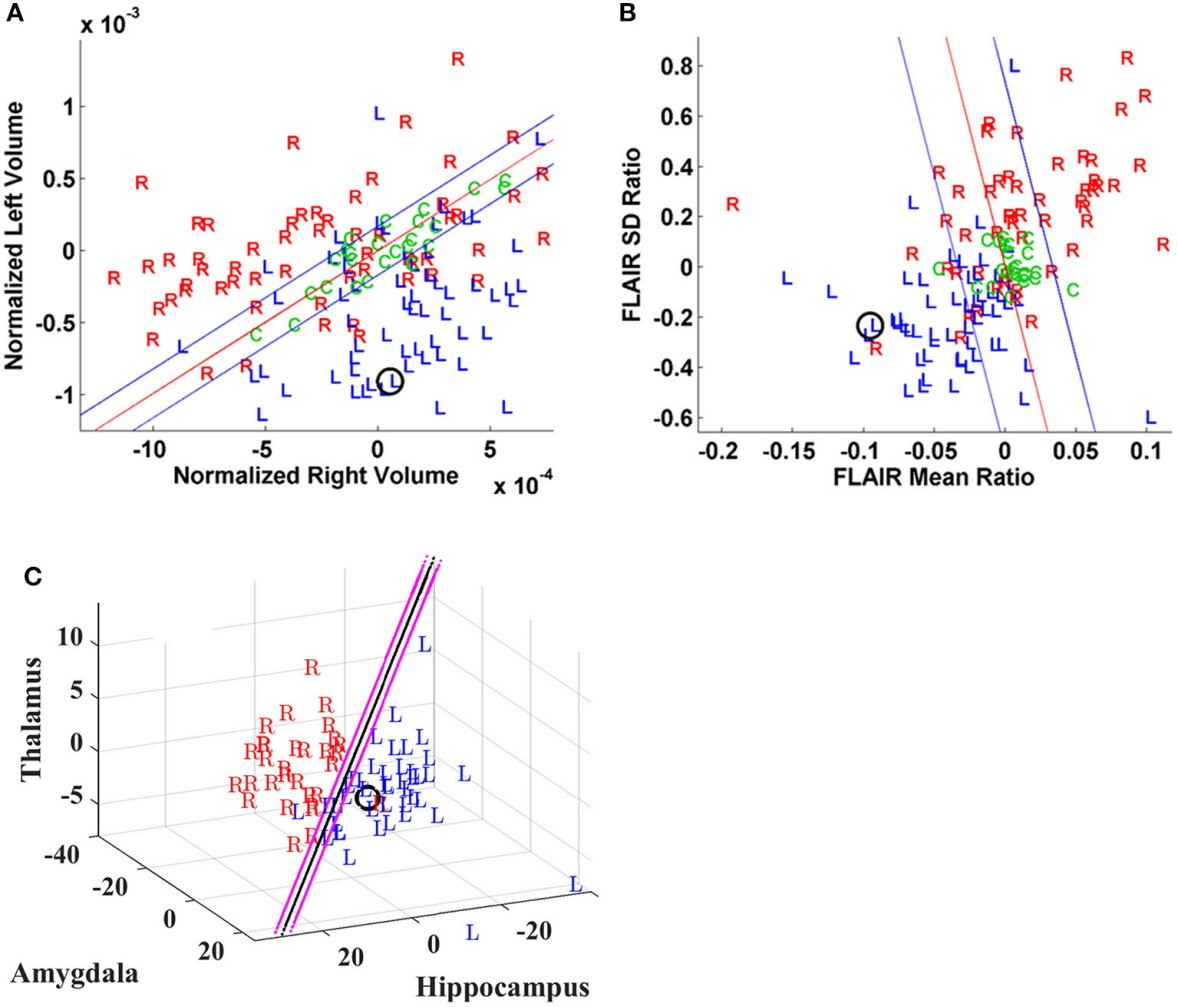
Case P15. Quantitative neuroimaging analysis in a patient who underwent Phase I study followed by a left temporal resection and achieved an Engel class I outcome. The epileptogenic site was predicted accurately by models for this case. The prospective case is shown (O). **(A)** Scatter plot of normalized hippocampal volumes showing distribution of control subjects **(C)** and study cases by their laterality for epileptogenicity (i.e., left vs. right). The volumes of control subjects are clustered and define the boundary domain (blue lines) within which the separation of a right- or left-sided mesial temporal lobe epilepsy (mTLE), to either side of the decision line (red line), may not be possible. **(B)** Scatter plot of mean and standard deviation ratios (right/left) of fluid-attenuated inversion recovery (FLAIR) MR signal intensity with control subjects **(C)** clustered in the boundary domain and study cases distinguished by their laterality for epileptogenicity (i.e., left *vs*. right). **(C)** The logistic decision plane of the multistructural analysis (MSA) model uses the normalized bilateral volume change [i.e., 100(vL – vR)/(Vl + vR)] of thalamic, amygdalar and hippocampal volumes to lateralize the prospective (O) case. Note that the decision plane is parallel to the angle of view; hence, it is seen as a line in two dimensions.

**Figure 2:**
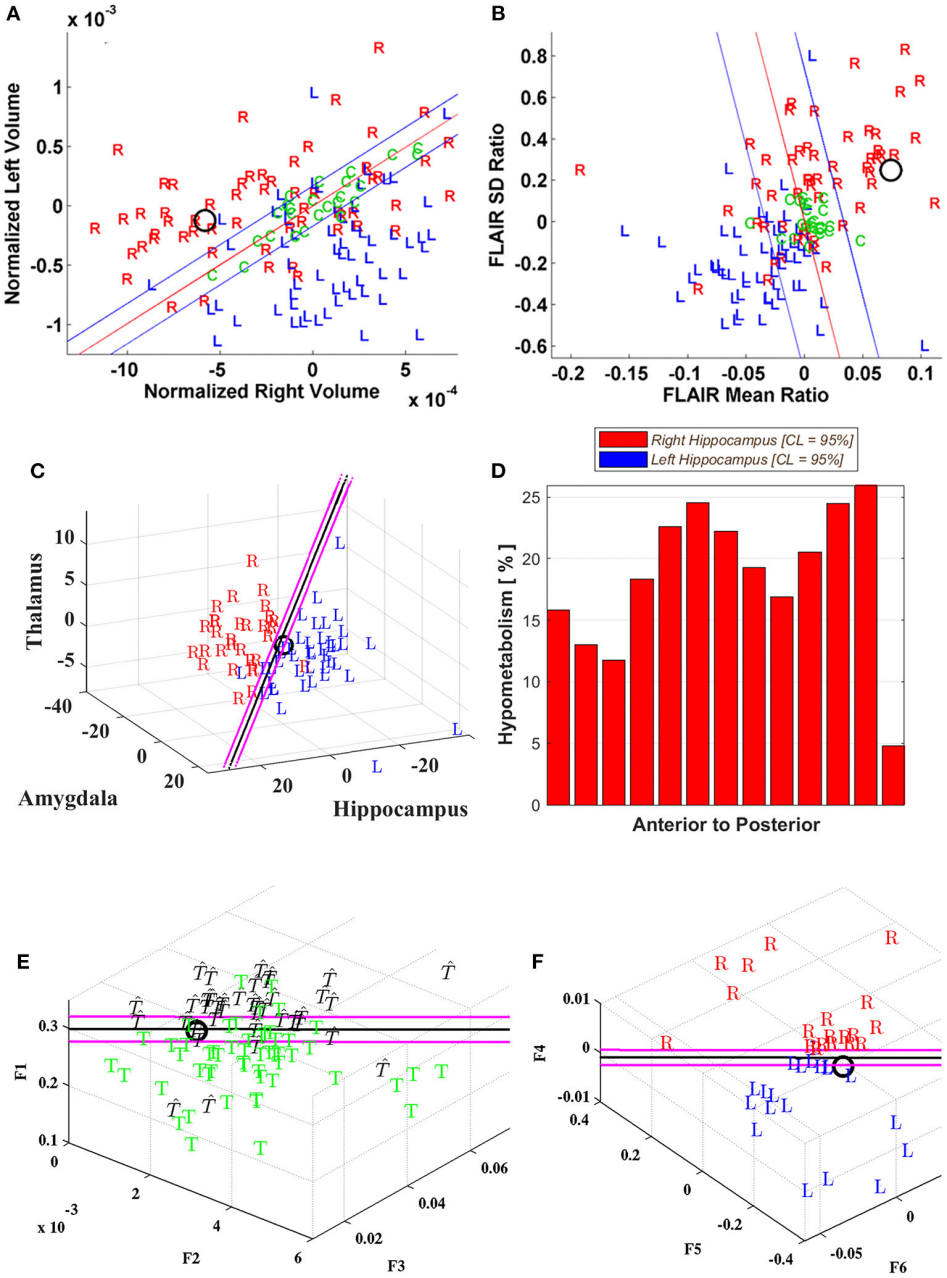
Case P03. Quantitative neuroimaging analysis in a patient who underwent Phase I study followed by a right temporal resection achieving an Engel class 2B outcome. The models did not adequately lateralize the epileptogenic side. Hippocampal volumetry **(A)** and FLAIR intensity **(B)** scatter plots lateralized this case as right-sided (O). However, the MSA model **(C)** was poorly lateralizing with the prospective case immediately to the left of the decision plane (i.e., UL; Table 1). **(D)** The cross-sectional position emission tomography (PET) profile of hippocampal hypometabolism strongly showed right-sided hypointensity for this case. Diffusion tensor image (DTI) connectivity analysis was indeterminate (i.e., U; Table 1). The support vector machine (SVM)-based classifiers developed for **(E)** categorizing whether the epileptogenic area resided in the temporal lobe, T, or not, T, and **(F)** lateralizing the epileptogenic area, showed the prospective case to reside within the boundary domain and on the borderline, respectively. Note that the decision plane is parallel to the angle of view; hence, it is seen as a black line in two dimensions. The uncertainty boundaries are demonstrated as pink lines which are defined here to be half the maximum margin of the support vectors (64). The features for categorization (F1–F3) and for lateralization (F4–F6) are defined in Methods section Diffusion Tensor Image Connectivity Analysis.

A Phase I study was judged sufficient for resection in seven cases (P03, 05, 07, 10, 15, 18, 47) by strict clinical determination, and of these, three cases (P03, 07, 18) did not attain an Engel class 1 outcome after 2 years although one (P47) did so after the first year. All had qualitatively determined MTS. In contrast to those cases which indicated clear predominance in laterality by quantitative metrics with Engel class 1 outcomes (Figure 1), the latter three showed more notable disagreement among metrics. P03, for instance, showed both hippocampal volume and FLAIR intensity metrics aligned ipsilateral to the side of the MTS; however, MSA (i.e., hippocampus, amygdala, thalamus) analysis favored the contralateral side and both DTI metrics were non-lateralizing (Figure 2). Although reduced hippocampal and multistructural volumes in P07 were identified ipsilateral to the side of the MTS, FLAIR intensity metrics fell well within the boundary domain. P18 showed reduced hippocampal volume on the right supported by MSA to a lesser extent (i.e., UR) but increased FLAIR intensity showed on the left with both rsfMRI and DTI connectivity and PET metrics also in support.

#### Phase II Study

A total of 34 patients underwent Phase II study, 17 (50%) underwent resection, 13 (38%) underwent RNS implantation, two (6%) underwent resection followed by RNS implantation and two (6%) deferred further intervention (see Table 1). MTS was reported in six cases (P12, 27, 31, 33, 43, 51) in this group but conflicting EEG and sodium amobarbital study data forced further Phase II study. Quantitative study in three of these six cases strongly confirmed agreement on the side of the MTS with four (P31) or five (P12, 27) quantitative attributes indicating the same. Two cases (P43, 51) showed mixed results with quantitative study. Both resection and RNS implantation was undertaken in P43 to effect an Engel class 1B outcome with hippocampal volume and DTI connectivity metrics identifying a left preference but MSA, DTI uncertainty and rsfMRI connectivity metrics favoring right. Likewise, in P51 with an Engel class 1A outcome, both hippocampal volumetry and FLAIR showed weak and opposing laterality yet MSA, PET, and MMM strongly supported the correct side. Of the 17 cases undergoing solely resection, an Engel class 1 outcome was attained in 13 (76%).

#### MTS Group

Of the entire group of 48 patients, 14 were reported to have an unequivocal unilateral MTS after imaging review. Six of these (P07, 12, 27, 31, 43, 51) underwent Phase II study. Of the MTS group, 11 (79%) underwent resection with three (P03, 07, 18) not attaining an Engel class I outcome. P33 was reported as showing bilateral MTS with greater involvement on the right as demonstrated by a reduced hippocampal volume. This was supported by MSA and MMM, favoring the side of the resection, although a contralateral slightly increased FLAIR intensity was identified (i.e., UL). A further breakdown of cases related to clinical complexity and outcomes is provided in the following subsections:

#### Non-MTS Group

**Figure 3:**
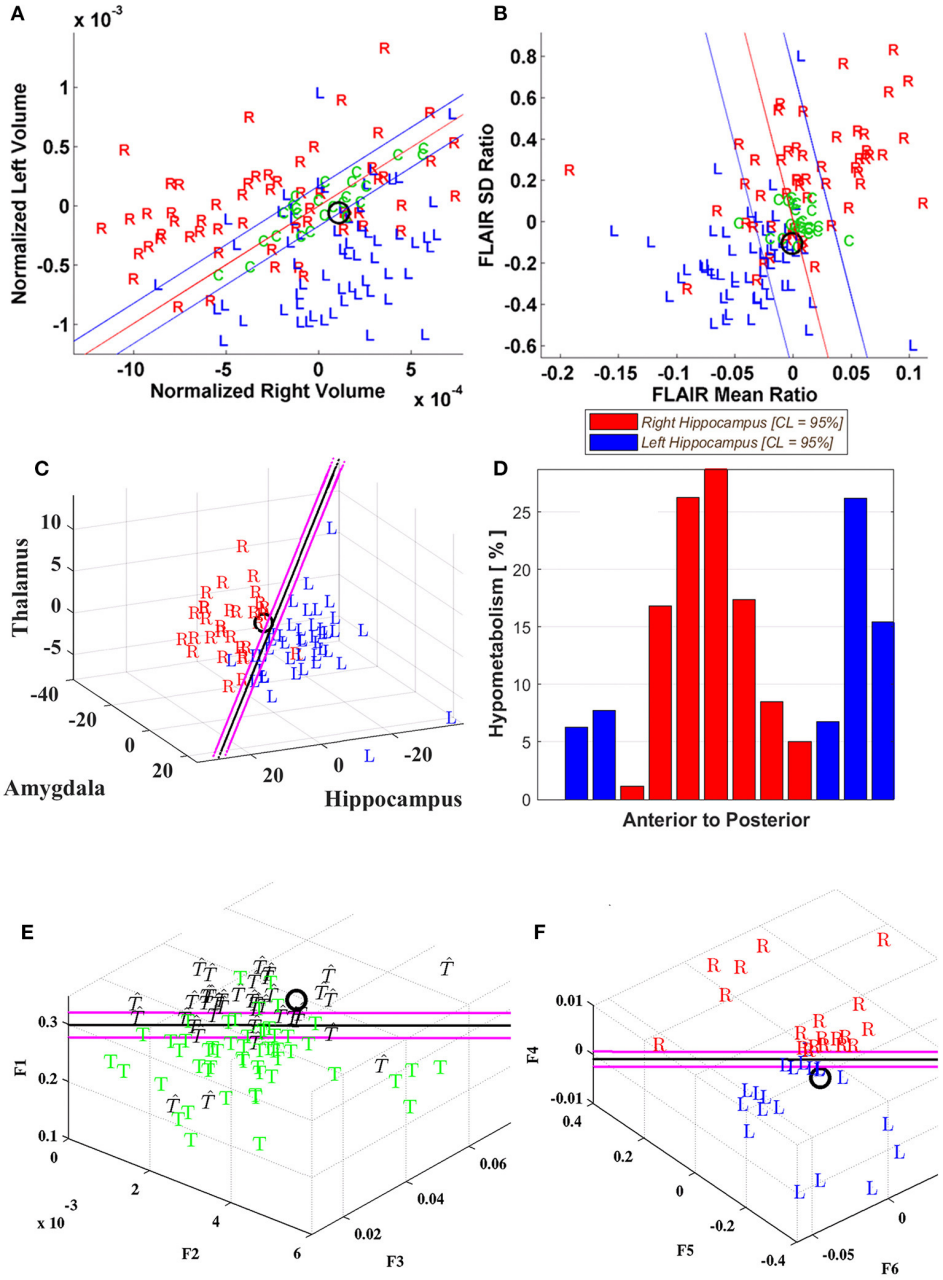
Case P20. Quantitative neuroimaging study of a patient who underwent both Phase I and II study followed by a right temporal resection and achieved an Engel class 1 outcome. Hippocampal volumetry **(A)** and FLAIR intensity **(B)** scatter plots showed poor lateralization (O) although MSA **(C)** identified a right laterality. PET **(D)** was indeterminate for the most part with variable slice-by-slice distribution favoring either right or left sides although predominating toward the right in the hippocampal body. DTI connectivity **(E, F)** favored the left minimally (i.e., UL; Table 1).

The non-MTS group comprised 33 cases of which 12 underwent resection and 14 required RNS electrode implantation within one or both cerebral hemispheres. Of those resected, nine (75%) attained Engel class 1 outcomes after 2 years. The remaining three cases (P01, 22, 42) showed poorer outcomes and, correspondingly, the quantitative analyses disagreed with the laterality in P01, showed a high degree of indeterminacy in P22 or a mix of the two features in P42, respectively. The others tended to show greater concordance among metrics but, in some, despite a favorable outcome, the variance caused interpretive problems and would not allow adequate lateralization (i.e., P17, 20, 48; Figure 3).

RNS electrode implants were lateralized to right or left sides in nine cases. The tendency for discordance among the metrics was greater than that found in the resected group but still showed predominance for the side of implantation in a few cases (P19, 24, 45). Among bilateral RNS electrode implant cases, much the same was apparent with greater variance among metrics and some degree of predominance of lateralizing features toward either side. Hippocampal volumetry was declared indeterminate in 60% of RNS cases.

#### Engel Class 1 Outcome

Seventeen patients achieved an Engel class 1 outcome following mesial temporal resection. Neuroimaging was assessed quantitatively using three to seven metrics (median, 6) that became available for analysis. Five patients showing the greatest discordance regarding their metrics were either identified possessing two to four metrics as indeterminate (i.e., ‘U'; P17, 20, 32, 48) or possessing opposite lateralities in a number of modalities (i.e., R vs. L; P29).

#### Engel Class ≥2 Outcome

Of the analyses performed in the entire study group of 48 patients, 26 (57%) had at least five quantitative imaging metrics completed and another 14 (30%) had four metrics completed. Of this group of 40 patients, 22 (55%) underwent resection of the putative site of epileptogenicity. An Engel class 1 outcome was not attained after 2 years in five patients (P03, 18, 22, 33, 42). Three of these underwent Phase II study (P22, 33, 42). Four to eight metrics were available to judge laterality in the five cases with notable disagreement in some (i.e., L vs. R; P18, 42) or mixed weak lateralization (i.e., UR, UL) and indeterminacy (i.e., U) among a number of metrics characterizing others (P03, 22). A notable example was P22 showing no MTS by report although Phase I and II studies confirmed right mesial temporal ictal onset. A right mesial temporal resection resulted in an Engel 2B outcome after 2 years. Quantitative studies all showed indeterminate or weakly lateralizing results for TLE except for the DTI uncertainly analysis which favored the left. Later MRI identified evolution of a right inferior precuneal lesion found histologically to be a combined pilocytic astrocytoma and cortical dysplasia. Intraoperative electrocorticography confirmed abundant epileptogenic activity and the site was laser-ablated with resolution of the epilepsy.

### Individual, Multistructural, and Multimodal Model Evaluation

To judge the value of quantitative analysis, both individual and cumulative (i.e., MSA, MMM), used in this study as a tool for providing lateralizing information, each metric was assessed by outcome category following resective surgery. Actual outcomes (i.e., Engel class 1, Engel class ≥2) were matched with those predicted by each of the metrics in the current study. Variable numbers of cases qualified for each of these attributes depending upon their actual use at the time of patient assessment and successful completion of the study. Of the 24 cases undergoing solely resection, for instance, all were available for both hippocampal volume and MMM analysis. Case numbers fell to 13 and 14 for DTI uncertainty and connectivity measures, respectively, and to insufficient numbers for resting state fMRI connectivity.

The best prediction of favorable outcome was achieved with MSA, MMM, and PET (Table 3). With MSA, 17 of 24 cases had an Engel class 1 outcome and a positive prediction of the correct side was attained in 15 cases (88%). Otherwise, for those cases resulting in poorer outcomes, the predicted side was opposite to the side operated in four (P01, 03, 18, 42) of seven cases (57%) and therefore in disagreement with clinical decision-making. Multimodal modeling proved also reliable with 14 of 17 cases (82%) showing an Engel class 1 prediction; it was indeterminate in two of these (P20, 48) and incorrect with laterality in one (P29). In the 11 cases with an unequivocal unilateral MTS, MMM lateralized eight (73%) that attained an Engel class 1 outcome. In two of the MTS cases with poorer outcomes, both MSA and DTI metrics identified contralateral features supporting their inclusion in a future model. Of a total of seven cases with poorer outcomes, MMM lateralized incorrectly in four (P03, 07, 18, 33) agreeing with the side operated. It disagreed with the side operated in one (P01) and was indeterminate in two (P22, 42). With PET profile analysis, of 12 cases with an Engel class 1 outcome, 10 were given a positive prediction of the correct side (83%). It incorrectly identified laterality in one (P16) and was indeterminate in the remaining case (P20). In the three cases with poorer outcomes, two were incorrectly lateralized (P03, P18) and one was shown to be indeterminate (P22). In the 20 cases for which FLAIR signal analysis was possible, 9 of 13 (69%) with Engel class 1 outcomes were correctly identified. A definitive ‘left' or ‘right' designation (i.e., scatter plot, >2sd) was possible in seven of the nine cases with MTS not declared in four of these. Of the seven cases with poorer outcomes, FLAIR signal analysis identified the side opposite the resection in two (P01, 33) and was indeterminate in two (P07, 22). Hippocampal volumetry provided a similar result by matching favorably in 13 (76%) of 17 cases with an Engel class 1 outcome in which a definitive “left” or “right” designation was provided in eight. One case (P17) was declared indeterminate as it was with MSA and both DTI measures. Otherwise, in three (P01, 18, 22) of seven cases with an Engel class ≥2 outcome, there was disagreement with the side operated. The DTI uncertainty analysis was applied to 14 cases of which 10 attained an Engel class 1 outcome with six (60%) correctly identifying the resected side while the DTI connectivity analysis was applied in 13 cases of which nine attained an Engel class 1 outcome with five (56%) of these matching the resected side. Three cases with poorer outcomes identified indeterminacy in the uncertainty analysis in two (P03, 18) and in the connectivity analysis in two (P03, 22).

**Table 3:** Match of predicted values of individual, multistructural and multimodal study metrics with actual outcomes (Engel class 1 vs. Engel class ≥ 2).

**Criteria**	**(i) Vol Ratio**	**(ii) FLAIR**	**(iii) MSA**	**(iv) PET**	**(v) MMM**	**(vii) DTI Uncertainty**	**(vii) DTI Connectivity**	**(viii) fMRI Connectivity**
Engel 1	13/17 = **0.76**	9/13 = **0.69**	15/17 = **0.88**	10/12 = **0.83**	14/17 = **0.82**	7/10 = **0.70**	5/9 = **0.56**	2/2 = **1**
Engel ≥2	4/7 = **0.57**	3/7 = **0.43**	3/7 = **0.43**	2/3 = **0.67**	4/7 = **0.57**	0/3 = **0.00**	1/3 = **0.33**	-
		**Engel class 1 Outcomes**			**Engel class ≥ 2 Outcomes**			
	**R** **\** **M**	**L&UL**	**R&UR**	**U**	**L&UL**	**R&UR**	**U**	
**Hippocampal Volume**	**L**	6	1	0	2	2	0	
	**R**	2	7	1	1	2	0	
**FLAIR**	**L**	4	1	1	2	1	1	
	**R**	1	5	1	1	1	1	
**MSA**	**L**	5	2	0	1	2	0	
	**R**	0	8	1	1	2	0	
**PET**	**L**	4	0	0	1	0	0	
	**R**	1	6	1	0	1	1	
**MMM**	**L**	5	1	1	2	1	1	
	**R**	0	9	1	0	2	1	
**DTI (u)**	**L**	5	0	0	0	0	1	
	**R**	0	1	4	1	0	1	
**DTI (c)**	**L**	2	0	1	0	0	1	
	**R**	1	3	2	0	0	2	

*Note: each row in the upper display shows the number of cases predicted in each category leading to the identified outcome so that for Engel class ≥ 2, the number of wrong-side predictions are provided. Comparative tables (Engel class 1 vs. Engel class ≥ 2) show outcomes achieved by resection (R) following standard clinical protocol decision-making (left vs. right) and by model prediction (M) for each attribute, indicating absolute or partial determinacy for the epileptogenic side (L & UL vs. R & UR)*.

The discrepancies toward laterality among the quantitative metrics in cases with poorer Engel class outcomes become evident when compared with those attaining Engel class 1 outcomes (Table 4). The mean value obtained for percentage agreement with laterality between standard clinic decision-making and individual metrics was notably greater among those with Engel class 1 outcomes than those without (i.e., 0.78 and 0.48, respectively; *p* = 0.0216). Considering only those cases in which Engel class ≥2 was achieved, relatively few cases can be assembled to establish specificity for each attribute. An indeterminate (i.e., U) allocation for any of these applications would render a definitive rejection of laterality. Otherwise, an allocation of laterality in one or more applications opposite to others might raise caution.

**Table 4:** Agreement of laterality among neuroimaging metrics with results of standard clinical decision-making in cases with Engel class 1 vs Engel class ≥ 2 outcomes.

**Patient ID**	**No of Metrics**	**Agreement (%)**
**Engel class 1 outcomes**		
5	4	100
10	7	86
11	4	100
12	6	100
15	6	100
16	5	80
17	6	33
20	7	29
27	6	83
29	7	43
30	7	86
31	4	100
32	6	50
44	3	100
47	6	100
48	6	50
51	5	80
Mean ± STD	5.6 ± 1.2	77.6 ± 25.9
**Engel class ≥ 2 outcomes**		
1	4	0
3	7	57
7	4	75
18	8	63
22	7	14
33	4	75
42	4	50
Mean ± STD	5.4 ± 1.8	47.7 ± 29.5

*A total of 17 cases with Engel class 1 outcomes and 7 cases with Engel class ≥ 2 outcomes are presented with their corresponding number of metrics applied in each case. The percentage agreement between the clinical decision of laterality and the number of metrics agreeing with the same laterality is provided, showing significant discrepancy between the groups (two-sample independent t-test, p = 0.0216)*.

### Responsive Neurostimulation

Fifteen patients underwent placement of an RNS unit with two also undergoing additional resection. All had attained at least a 50% reduction in seizure frequency within a three-year time period with two having exceeded 80%. Six of the 15 had bilateral implants with all showing features of bilateral disturbance electrographically and, four of these, an absence of hippocampal volume asymmetry (i.e., U). For all patients undergoing RNS implantation, nine had shown no volume asymmetry with three others declared as definitively lateralized as right or left. Of the nine patients undergoing unilateral RNS implantation on the right (3) or left (6), only three showed some indication, by neuroimaging attribution, of ipsilateral abnormality whereas the others showed a mix of results insufficient to declare a trend.

## Discussion

The findings of this study lend support to a neuroimaging-based decision-making process that seeks to determine laterality of a putative mTLE or exclude a pure unilateral temporal lobe epileptogenicity (i.e., bitemporal, extrahippocampal, extended extratemporal or pure extratemporal). Both MSA and PET profile analyses, with Multimodal Model (MMM) analysis followed by FLAIR signal analysis and hippocampal volumetry provided means of lateralizing a mTLE sufficient to proceed to a favorable outcome with resection. The MMM decision scheme used here included both MSA and PET as indices, adding a multistructural and a functional element, respectively, to its decision-making paradigm. It also contained SPECT as a metric which was not used in the current analysis of prospective cases. The use of other attributes in the study may well have provided even better predictions. Those cases in which there was considerable variability in lateralization (i.e., R vs. L) among the metrics, mixed with some weakness in lateralization (i.e., UL, UR) or simply, indeterminacy (i.e., U), resulted in a poorer outcome (Engel class ≥2) when undergoing resection. Optimization of the approach is still required and can be achieved through the selection of neuroimaging attributes better able to discriminate those features necessary for decision-making. Consideration must be given to weighting some attributes over others based upon their ability to discriminate and then to combine them into a model that can be tested.

Several multistructural lateralization studies of mTLE (1, 54, 62, 64, 66, 67) have shown utility in providing reliable measures of laterality. More recently, using data-mining methodology in a retrospective study, Mahmoudi et al., confirmed the optimal number of neuroanatomical sites for this purpose to include the hippocampus, thalamus, and amygdala, and provided a model by which to establish laterality using a scatter plot (62). The tristructural volumetric application proved effective in lateralizing 98.5% of cases compared with that of an 82.4% lateralization using hippocampal volumetry alone. Moreover, MSA had correctly lateralized 92.9% of MTS-negative mTLE cases. This is borne out in the current prospective study with MSA again outperforming hippocampal volumetry alone. The MMM approach in the current study included both biomarkers and, under these circumstances, an argument can be made to exclude hippocampal volumetry from such a model in favor of another more opportune attribute such as DTI connectivity.

The hallmark imaging feature of mTLE is MTS in which both hippocampal volumetry and FLAIR mean signal intensity combine to provide some probability of epileptogenicity sufficient to lateralize a mTLE in several cases. In the current study, hippocampal volumetry alone correctly lateralized 76% of resected cases whereas combined FLAIR mean signal and standard deviation measures did the same in 69%. Twenty resected cases had both measures completed, although five cases had one of the two measures declared indeterminate. In 11 of the 20 cases, an MTS was not declared although nine attained an Engel class 1A outcome but only five of these were confirmed by one or both metrics. Such findings align with recent determinations of the predictive value of both hippocampal volumetry (67%) and hippocampal FLAIR mean signal and standard deviation (70%) (10) and point to shortcomings in the use of this dual measure.

A neuropathological analysis of cases was not consistently performed in this series to warrant inclusion and to offer some insight into associated histopathological features; however, in a prior study (10), such analysis of an mTLE caseload identified Ammon's horn sclerosis in 21/31 cases (68%). Interestingly, some qualitative differences were identified histologically wherein gliosis predominated without notable cell loss in cases of MTS (10, 22).

Interictal PET study with 2-[^18^F]fluoro-2-D-deoxyglucose (FDG) has been useful in identifying epileptogenic sites often prior to structural changes such as MTS (68–70) although metabolic abnormalities can often be found at more than one site, sometimes complicating the localization of primary nodal sites of epileptogenicity (71, 72). Moreover, distinct hippocampal volume asymmetries in mTLE cannot be ascertained in 15–30% of cases (73). Kerr et al. showed FDG-PET to accurately lateralize epileptogenicity in 89% of patients (74). A prospective study of 23 patients with age-matched controls also lateralized 87% of TLE cases with FDG-PET where hippocampal volumetry did so in only 65% (48); however, only hippocampal volumetry was predictive of an Engel class 1 outcome. Binding of [^11^C]flumazenil (FMZ) to GABA_A_ receptors with PET imaging provides a more direct ligand-related means of detecting abnormality in TLE and may afford further utility in investigating mTLE. Hammers et al. identified 16 of 18 (90%) patients with mTLE despite a normal MRI, characterized by quantitative volumetric and T2 signal intensity measures, to have functional abnormalities with FMZ-PET (71). The current study supports the use of PET as a suitable metric in a multimodal decision-making scheme for lateralization and possibly for localization of an mTLE, adding favorably to the results obtained with MSA in such a model.

Multimodal postprocessing is defined as the simultaneous rendering of various spatially coregistered modalities, both structural and functional, for the purpose of identifying localizable abnormalities (52). A multimodal computer-aided lateralization framework would ostensibly increase sensitivity and confidence in lateralizing mTLE. The scheme used in the current study provides evidence of the utility of this approach. These modalities differ in their reliabilities and may, to some degree, show discrepancies in predicting laterality. Hence, simply combining data from multiple modalities will not necessarily enhance accuracy. Optimizing a battery of select neuroimaging attributes in such a way as to avoid the curse of dimensionality would provide an ultimate solution to this problem. In a previous study (51), 10 univariate or multivariate response-driven lateralization models were developed using MRI, DTI, and SPECT attributes and logistic regression, to determine the side of epileptogenicity in TLE patients. By incorporating all multivariate attributes for 138 TLE cases that had at least one imaging attribute and imputing the mean value of the measured attributes of the control cases into the corresponding missing attributes, an all-inclusive model reached a probability of detection of the epileptogenic side of 0.83. This response model allowed the epileptogenic side to be detected in 90% of TLE patients. A high reliability for lateralization could be established by incorporating conventional (i.e., MSA, FLAIR, MRI), functional (i.e., PET, rsfMRI) and microstructural (i.e., DTI) attributes into a single MMM analysis that would likely improve upon the analysis put forward here.

The use of SPECT was excluded from this study because of a lack of availability during the timeframe of the study. It constituted part of the metrics ensemble within the MMM set established remotely that was available for comparison. The application of SPECT as an assessment of blood perfusion for lateralizing and localizing epileptogenicity has been shown to be reliable in several studies (35, 75–77). A retrospective study of hippocampal subtraction ictal SPECT in 48 patients with an Engel class 1A outcome showed a lateralization accuracy of 91%, slightly higher than that achieved with FLAIR MR signal analysis in the same study (77). Its use in further studies of this sort would be of interest.

Because DTI measures provide a sensitive tool for detecting microstructural changes in brain tissue often before any abnormality appears on structural MRI (78, 79) and the widespread propagation of synchronized neuronal firing in mTLE affects a number of remote structures (80), they become a useful tool for identifying change in the integrity of white matter fiber tracts involved in mTLE (81, 82). A retrospective study with DTI in mTLE also showed promise in distinguishing both bilateral from unilateral cases and right from left mTLE cases by assessing fractional anisotropy and mean diffusivity in the cingulum, the forniceal crura and corpus callosum (23). It succeeded in differentiating 54 cases into right mTLE, left mTLE, and bilateral mTLE from control. The current prospective study showed some utility of both connectivity and uncertainty measures of DTI although with reduced case numbers in comparison with the other metrics. Of some interest are the indeterminate features that may provide some warning regarding laterality of an mTLE or its centrality as a principal node in the epileptogenic network. More study is required with greater numbers of cases for an adequate conclusion to be drawn.

This study is based on the presumption of dealing at the outset with what may possibly be a TLE. Although experience has shown that, in fact, the majority of cases operated upon present with a pure mTLE that can be effectively treated by surgery, several do present with epileptogenic networks extending beyond the confines of the temporal lobe to involve extratemporal sites as immediate regional extensions within the neighboring insula, frontal cortex, or parietal cortex, or are more remotely linked as with the cingulate gyrus (i.e., P46) or precuneus (i.e., P22). Other cases may be identified at the outset as bitemporal epilepsies behaving as independent epileptogenic networks or as linked entities. Some, of course, are purely extratemporal. Much of this accounts for the failure of resective surgeries targeting a putative mTLE when electrographic study was suggestive but inconclusive and qualitative neuroimaging was supportive. Part of the aim of having a concerted effort of investigation centered upon a quantitative neuroimaging platform must be not only to distinguish the features of a distinctive localized network as in the case of an isolated mTLE but to provide strong indication of why it may not necessarily be the latter.

A computer-aided quantitative multimodal multistructural response model, using a preferred list of MRI and nuclear medicine-derived attributes, shows promise in optimizing the lateralization of mTLE and the selection of surgical candidates and possibly reducing the need for Phase II study. The approach overcomes the intrinsic limitation of individual modalities by increasing the information content made available (52). Phase II evaluation itself may fail to survey the entire epileptogenic network sufficiently to declare its full extent. A preliminary quantitative multimodal approach provides the opportunity to identify most nodal elements that designate a significant component of the network well enough that, in many circumstances, resection of a contained segment may succeed in a seizure-free status. The results of this study with its limited cohort emphasize the concerns that are commonly raised in patients in whom both EEG analysis with Phase I and II and qualitative imaging interpretations taken in combination can lead to a wrong conclusion. The degree to which there is disagreement among the various quantitative metrics must be considered a parameter by which we judge whether epileptogenicity exists in a single temporal lobe, particularly, its mesial aspect.

As a single institutional prospective comparative study involving multivariate analysis of neuroimaging features, this work has its limitations. The nature of this sort of study with all data accrued from a single institutional experience limits the number of cases that could be gathered within a given timeframe, largely because of the unpredictability of a prospective analysis and the number of comparative neuroimaging methods employed. Moreover, data inhomogeneity arises when using clinical standard-of-care protocols as part of daily operations so that not all patients will necessarily undergo all investigations. However, the strength of the work is that the present study is of a single institutional nature as was the quantitative analysis itself. This provides assurance that the standards of clinical decision-making, outcomes and the analyses were performed uniformly across the entire study.

The creation of a standardized quantitative neuroimaging platform that incorporates multistructural and multimodal attributes coupled with initial (i.e., Phase I) video-EEG investigation may limit the need for subsequent more invasive (i.e., Phase II/III) study by declaring a unilateral mesial temporal epilepsy more objectively or limit the targeting of intracranial sites when there is further need of such study. Apart from ensuring greater patient safety and improving upon the efficiency of investigation, the expense is likely to be reduced as improved machine-learning methods are used with such platforms for decision-making purposes (83). This prospective study of a variety of quantitative neuroimaging applications for the lateralization of a putative TLE provides a comparison with the benefits of each. Certain applications focused upon combined multistructural and multimodal attributes hold promise in decision-making. The study supports future directions in the use of machine learning and decision-support platforms in defining laterality and the likelihood of success with epilepsy surgery as it applies to the temporal lobe (84, 85). Specifically, the presence of agreement among individual and combined neuroimaging metrics regarding laterality points to a high degree of assurance of a specific site of epileptogenicity. By contrast, greater numbers of these metrics showing a discordance of laterality or indeterminacy indicates an absence of a distinct laterality and the likelihood of a poorer outcome should resection be undertaken.

## Data Availability Statement

The original contributions presented in the study are included in the article/supplementary material, further inquiries can be directed to the corresponding author/s.

## Ethics Statement

The studies involving human participants were reviewed and approved by Institutional Review Board (IRB) of the Henry Ford Health System (HFHS); IRB of Spectrum Health (SH). Written informed consent to participate in this study was provided by the participants' legal guardian/next of kin.

## Author Contributions

KE did the conceptualization, data curation, formal analysis, investigation, validation, and writing. ED-B handled the data curation, formal analysis, investigation, methodology, and validation. JH also handled methodology, resources, and writing. HS-Z also did the conceptualization, handled the funding acquisition, and project administration.

## Funding

Funding will come through the Department of Clinical Neurosciences, Spectrum Health.

## Conflict of Interest

The authors declare that the research was conducted in the absence of any commercial or financial relationships that could be construed as a potential conflict of interest.

## Publisher's Note

All claims expressed in this article are solely those of the authors and do not necessarily represent those of their affiliated organizations, or those of the publisher, the editors and the reviewers. Any product that may be evaluated in this article, or claim that may be made by its manufacturer, is not guaranteed or endorsed by the publisher.
